# Supplementary material for: Resistance training leading to repetition failure increases muscle strength and size, but not power-generation capacity in judo athletes
Source: PLoS One. 2024 Aug 28;19(8):e0307841. doi: 10.1371/journal.pone.0307841 (PMC11356431; doi:10.1371/journal.pone.0307841)
Supplement: S2 File — (PDF) [file pone.0307841.s002.pdf]

別紙様式２（第８条関係）

審 査 結 果 通 知 書

Notification of Examination Results

第１１－１０２号
令和元年１１月２６日
26. Nov. 2019

高井　洋平　　様
Yohei Takai

倫理審査小委員会委員長　印
Ethics Review Subcommittee Chairperson

課題名：異なる力発揮様式によるトレーニングが筋の形状および筋機能に与える影響に関する研究
A Study of the Effects of Training with Different Exercise Modes on Muscle Size and Function

責任者：高井　洋平
Yohei Takai

実施計画を令和元年１１月２６日の委員会で審査し、下記のとおり判定したので通知します。

判　定　結　果	（１）承　認　　（２）条件付承認　　（３）計画変更の勧告<br>Approval<br>（４）不承認　　（５）非該当
判　定　の　理　由	

【研究活動における発明等に関する注意】
通常の研究活動及び企業との共同研究等において発明等が生じた場合は、速やかに学術図書情報課研究支援係（内線 4878）へご相談願います。
なお、特許出願前に学会、論文等で発表を行った場合、新規性が失われて特許を受けることができなくなる可能性もありますのでご注意下さい。
