# Supplementary material for: Resistance training leading to repetition failure increases muscle strength and size, but not power-generation capacity in judo athletes
Source: PLoS One. 2024 Aug 28;19(8):e0307841. doi: 10.1371/journal.pone.0307841 (PMC11356431; doi:10.1371/journal.pone.0307841)
Supplement: Attachment — Submitted filename: PLOS ONE_Nakatani_JudoTR_Response to Reviewers_submit.docx [file pone.0307841.s003.docx]

**Response to comments of Reviewer #1**

**Review Comments to the Author**

In this study neuromuscular adaptations to three 6-week elbow flexor training programs differing in intensity of load and muscular failure (30% 1RM leading to failure, 30% 1RM not lading to failure, and 80% 1RM performed to failure) were contrasted in a small sample of judo athletes. I have the following concerns about this manuscript:

1. Sample size of each group is very small (between 6 and 10)

2. The authis must provide a detailed description of all the procedures.

3. Statistical analysis should be improved (see specific comments)

4. Discussion section is too long, being similar to a narrative review. In my opinion it should be rewritten to be focused on the main findings of the results.

**Response**

Very thanks for your consideration and valuable suggestions to improve our manuscript. We read the reviewer’s comments very carefully and made the following changes in accordance with the comments and suggestions of the reviewer. The revised parts in the manuscript were highlighted by describing the page numbers and line numbers in our response to each of your comments. We hope that the revised manuscript answers the questions and comments of the reviewers.

***SPECIFIC COMMENTS (L= Line)***

#1. **L50. I´d suggest using the term heavy-load and light-load instead heavier/lighter-load.**

- According to the comment, we have done in the revised text. (L36-37, 48, 59-60, 62, 72-73)

#2. **L61-65. This statement is not true. There are several studies that show how RT not leading to muscular failure optimizes power gains.**

- We have modified the corresponding part. Two studies have investigated the effects of RF and NRF training on power in athletes. The previous findings indicate that while improvements in muscle strength and power in elite junior athletes were greater with RF compared to NRF (22), those in 1RM and power in basque ball players were similar between NRF and RF after 11 weeks of resistance training (23). Thus, there is no consensus among the previous studies. To avoid misleading readers, the following correction is made. (Page 4, L65-66)

“A few studies have examined the effect of resistance training leading to repetition failure on power generating capacity in athletes.”

#3. **L85-86. The authors should reword this sentence.**

- The corresponding part has been removed in the process of revision. (Page 5, L85)

#4. **L102-107.The wording of this paragraph is confusing to me. I would suggest reviewing it. On the other hand, what do you mean by ballistic? Throwing the load? At maximum voluntary velocity? Wasn´t maximal intended velocity performed with 80% 1RM load?**

- We added a note that we consider ballistic to be a voluntary maximal speed contraction. We have changed the sentence of L101-105 to the following sentence.

“Three training programs were designed to combine load (30 or 80% of 1RM) and fatigue condition (repetition failure or not repetition failure). The detail of the training program was described later. In the two programs with light load, the participants were asked to conduct concentric phase with ballistic contraction (at maximum voluntary velocity contraction) mode.”

- In addition, the phrase "as fast and **ballistic** as possible" in the RF_LB_ (L144~149) and NRF_LB_ (L156~161) paragraphs was changed to "as fast and **forceful** as possible" in order to convey the "ballistic" behavior more clearly. (Page 9, L144 and Page 10, L157)

#5. **L 110. This period corresponds to COVID-19 pandemic breakdown, doesn´t it? Were the participants following their usual training program? On the other hand, the sample size is quite limited and should be commented a s a limitation.**

- As pointed out, our study period coincided with the COVID-19 pandemic. Since the progression of the pandemic was slower in our region where we conducted this study compared to urban, there was no need to halt the training intervention. Therefore, all participants were able to complete the training program. On the other hand, we have understood that the sample size was small, and we would have planned to conduct additional experiment. Unfortunately, our region also experienced a pandemic surge during the planned additional experiment period, preventing us from conducting the additional experiments. We have added to the limitation about the small sample size. (Page33, L468-469)

“Fourth, the study had a relatively small sample size and thus may have been somewhat underpowered to detect significant changes between groups in certain outcomes.”

#6. **L112-116. Please specify the description of the sample: did they perform resistance training? In this case, what was training frequency? Did they include the tested exercise in their training program?**

- In response to your suggestion, we have made the following modifications. (Page 7-8, L114-116)

“Judo training and resistance training were performed 5-6 and 2-3 days per week, respectively. During the experimental period, resistance training targeting the elbow flexors was not performed.”

#7. **L147. Please, replace are by were.**

- We have done. (Page 9, L144)

#8. **L 147150. It is strange that the athletes were able to maintain this pace until muscular failure. It is well known that velocity decreases with fatigue, so it is very strange to perform repetitions until muscular failure without losing velocity. Why RFHT was not performed to the maximum intended velocity? How could this have affected your results?**

**See for example:**

[**https://www.ncbi.nlm.nih.gov/pmc/articles/PMC9613575/**](https://www.ncbi.nlm.nih.gov/pmc/articles/PMC9613575/)**;**

[**https://pubmed.ncbi.nlm.nih.gov/8444715/**](https://pubmed.ncbi.nlm.nih.gov/8444715/)

- RF_HT_ raised the dumbbell for 4 s and lowered it for 4 s, so they did not exert as fast and forceful as possible. They lifted the dumbbell by 8-10 times, but the tempo of lifting was constant. Furthermore, the reason we did not ask the RF_HT_ group to lift the weight as fast and hard as possible was due to protect the subjects from the risk of injury. Therefore, the exercise was performed at a constant tempo. Since there may be room for further investigation regarding the heavy-load training with ballistic contraction, we have added the following sentences to the limitations section. (Page 31-32, L437-440)

“RF_HT_ performed the exercise task with a constant tempo rather than the maximum intended velocity to protect the participants from the risk of injury. The effects of heavy load training with ballistic contractions on muscle strength and power are subjects in further investigation.”

#9. **L152-153. A high inter-subject variability exits regarding the maximum number of repetitions performed with and specific relative load, therefore 20 repetitions might have entailed different level of effort between participants. Did you test maximum number of repetitions with this load? If this was not the case, you should discuss this point as a limitation of your study.**

- We have not determined the maximum number of repetitions with a specific load. We have added the following sentences to the limitations section. (Page 32, L440-443)

“Furthermore, a high inter-subject variability exits regarding the maximum number of repetitions performed with and specific relative load, therefore 20 repetitions might have entailed different level of effort between participants of NRF_LB_.”

#10. **L163-165. I do not understand this point. This** **a surrogate estimation of mechanical impulse, isn´t it? On the other hand, RT with light loads was performed at maximum intended velocity, therefore time under tension varied throughout each set.**

- As pointed out, this is a surrogate estimate of mechanical impulse. We have removed the word "per session” from that sentence. In the light-load training employed in this study, participants used a metronome to adjust the eccentric phase to a tempo of 60 bpm and the concentric phase to as quickly a force as possible. Time was not measured during the concentric contraction phase of NRF and RF. Therefore, the contraction duration was less than 1 second, but the lifting time was assumed to be 1 second. We have modified it as indicated below. (Page 10-11, L165-170)

“In the present study, the training volume was defined as the product of absolute load (kg), total number of repetitions (rep), and total time required for concentric and eccentric phases (s). A metronome was used to describe the tempo during the exercises to 60 bpm. When participants could no longer maintain the tempo within 1 bpm, the training for each set was discontinued. In NRF_LB_ and RF_LB_ with ballistic contraction, the contraction duration was less than 1 second so that lifting time was considered to be 1 second.”

#11. **L249. How did you obtain angular velocity?**

- The angular velocity was obtained by a potentiometer mounting on the lever arm. The following sentences are explained in the chapter on testing protocols and are omitted here.

Experimental setup and procedures of the test protocols were the same as those adopted in a prior study [8]. (Page 11, L178-179)

**STATISTICAL ANALYSIS:**

#12. **L265. You had three groups and therefore one-way ANOVA should also be used for baseline measurements?**

**-** In the corresponding part, we have mistaken the description of the statistical analysis of group difference at baseline. As the reviewer pointed out, we have used one-way ANOVA with factorial and post hoc test for group comparison. We have changed the corresponding part to the following sentences. (Page 16, L270-272)

“We used a one-way analysis of variance (ANOVA) to examine group differences in the measured variables at baseline and to test the main effect of group on total training volume and total number of repetitions.”

#13. **L270-271. Sample size is really small, and therefore the use of non-parametric statistic should be recommended. Did you test normality and homocedasticity of your variables?**

- We have conducted the Shapiro-Wilk test for normality, and the test statistics, degrees of freedom, and p-values are presented in the table below. Normality is ensured for all data in the baseline. Mauchly's sphericity test was used for homogeneous dispersion, and Greenhouse-Geisser was used when sphericity was not satisfied.

Table. Normality tests

|  | Statistics | | |
| --- | --- | --- | --- |
| MT | 0.988 | Degrees of freedom | p-values |
| CSAindex | 0.984 | 23 | 0.99 |
| MVC | 0.935 | 23 | 0.957 |
| 1RM | 0.962 | 23 | 0.14 |
| RFDmax | 0.97 | 23 | 0.503 |
| F0 | 0.958 | 23 | 0.68 |
| V0 | 0.927 | 23 | 0.434 |
| Pmax | 0.96 | 23 | 0.094 |
| F-Vslope | 0.941 | 23 | 0.456 |

In addition, the statistical power (1-β) was computed with total sample size (N = 26), effect size calculated from partial η2 and the significant level (p<0.05). As the results, the statistical power was more than 0.8 in all variables. Therefore, we have considered that the experimental groups were sufficient to achieve the required statistical power. This point was important in this study, so we have added the description on the results of the power analysis to the discussion section. (Page 26, L329-333)

“It was doubtful that there is any power of detection because the sample size in this study was small. So, the statistical power (1-β) was computed with total sample size (N = 23), effect size calculated from partial η^2^ and the significant level (p<0.05) for 1RM, muscle size, MVC, F_0_/CSA_index_ and P_max_/CSA_index_. As the results, the statistical power was more than 0.8 in all variables. Therefore, the sample size may be sufficient to achieve the required statistical power.”

Table. Power analysis of main effect of time and group-by-time interaction.

| Variables | pη^2^  Time | 1-β = 0.8 | pη^2^  Interaction | 1-β = 0.8 |
| --- | --- | --- | --- | --- |
| 1RM | 0.88 | 1.00 | 0.19 | 0.97 |
| MT | 0.59 | 1.00 | 0.04 | 0.35 |
| CSA_index_ | 0.60 | 1.00 | 0.03 | 0.27 |
| MVC | 0.55 | 1.00 | 0.05 | 0.43 |
| F_0_/CSA_index_ | 0.28 | 0.99 | 0.06 | 0.50 |
| P_max_/CSA_index_ | 0.28 | 0.99 | 0.06 | 0.50 |

#14. **L 275. Please, report 95% confidence intervals for Hedge´s g.**

- Tables 1-3 (Page 19, 21 23-24)and corresponding sections have been updated. (Page 17, L279-280)

“We calculated Hedges’ g [31] with 95% confidence intervals as indices of effect sizes.”

#15. **L282-285.** **This result is trivial given the experimental design. On the other hand it highlights that differences or lack of differences may be caused by differences in volume instead of differences in loads. In my opinion this is the main limitation of this study since the independent variable is not well controlled.**

- We have reached a consensus. The following sentence was added to the limitation paragraph. (Page32-33, L449-467)

“Third, there are various training variables that contribute to increase muscle mass and strength, and it is possible that the effects of different conditions have either masked each other or created synergistic effects. Recent meta-analyses have concluded that for strength improvements, increases in strength are superior in high-load RT programs [20] and that multi-set protocols are more effective than single-set ones [61]. Training fast produces greater strength increases than training slow; however, there does not appear to be any additional benefit of training with both multiple sets and fast contractions [62]. Resistance exercise performed to failure elevates muscle protein synthesis regardless of training volume (sets × reps) or % one repetition maximum load [63, 64]. Furthermore, Sampson and Groeller [65] conducted a study where they varied the speed and number of repetitions in elbow flexion exercises, and reported that, despite differences in training volume, no significant differences were found in the effects on muscle mass and strength. They suggest that repetition failure and training volume may be of less importance for the development of muscle hypertrophy and strength when the characteristics of the muscle activation are manipulated. In this study, despite the fact that training volume differed among the three groups, both muscle strength and muscle hypertrophy increased similarly regardless of any of the groups. Similar skeletal muscle adaptations can be gained with rapid muscle activation in the absence of repetition failure and a concurrent reduction in the total exercise volume. Hence, we cannot rule out a possibility that the current results may differ if the training volumes are aligned.”

#16. **L290-292. Please note that figure 3A sems to show simple contrasts that are not supported on significant interactions. Pairwise comparisons that are not based on significant interaction should be removed throughout the manuscript. In this regard, discussion and conclusions must be properly adapted.**

- As the reviewer pointed out, the 2-way ANOVA showed that there were no interactions between group and time, indicating that the magnitude of the training effects was similar across groups. The significance markers shown in the figure represent significant main effects without interaction. Since the results of the main effects are crucial, the notation in the figure remains unchanged. We will remove it from the diagram as it may mislead the viewers. (Page 17, L292)

#17. **L312-314. Was there a tendency or a significant main effect of time? Please, clarify this point.**

- As shown in Table 3, there was a main effect of time (P = 0.01). We have modified it. (Page 22, L317-318)

“F_0_/CSA_index_ and P_max_/CSA_index_ had significant effects of time without significant interactions of time and group (Table 3, Fig 5).”

#18. **Table 3. Units of slope should be reported**

- The unit of the slope is “N/ms”. We have done. (Page 23, Table 3)

**DISCUSSION**

#19. **Discussion is too long considering your findings: statistically speaking, the lack of interaction suggest that training conditions induced similar adaptations, despite the differences in volume. In my opinion the discussion must be rewritten and focused on this point. Finally, the interpretation of your results from the perspective of differences in volume should not be limited to conclusions.**

- We have agreed and removed some paragraphs from the discussion (Page 27, L346; Page 28, L374;).
