# Supplementary material for: Resistance training leading to repetition failure increases muscle strength and size, but not power-generation capacity in judo athletes
Source: PLoS One. 2024 Aug 28;19(8):e0307841. doi: 10.1371/journal.pone.0307841 (PMC11356431; doi:10.1371/journal.pone.0307841)
Supplement: Attachment — Submitted filename: PLOS ONE_Nakatani_JudoTR_Response to Reviewers_submit2.docx [file pone.0307841.s004.docx]

**Comment #1**

Lines 64-65: “A few studies have examined the effect of resistance training leading to repetition failure on power generating capacity in athletes.” Please provide some references to support this statement.

**Response**

We have added the relevant references to the corresponding part. (Page 4, Lines 64-65)

“A few studies have examined the effect of resistance training leading to repetition failure on power generating capacity in athletes [19, 20].”

**Comment #2**

Line 106: Please replace "was" with "is." On the other hand, was the exercise with heavy load performed at submaximal velocity? Please clarify this. Intended velocity is a key point for specific adaptations, and thus, the authors should consider this aspect in the discussion section.

**Response**

We have done. (Line 102)

The text has been changed as follows: (Page 7, Line 102-105)

“The detail of the training program is described later. In the two light-load programs, the participants were asked to conduct concentric phase with ballistic contraction (maximum voluntary contraction velocity) mode. On the other hand, the participants involved in the high-load program performed the exercise task at a constant tempo.”

The tempo of the exercise with heavy load was constant, two seconds in the concentric phase and 2 seconds in the eccentric phase. This has been addressed in the section of “Training programs” (Line 153). Additionally, the participants involved in RF_HT_ performed the exercise task with a constant tempo rather than the maximum intended velocity to protect the participants from the risk of injury. The effects of heavy load training with ballistic contractions on muscle strength and power are subjects in further investigation. When the reviewer commented last time, we have added this point to the discussion section. (Lines 436-439)

Additionally, we have removed the sentence with the same intent as above. (Page7, Lines 106~)

~~“In the two programs with light load, the participants were asked to conduct concentric phase with ballistic contraction mode.”~~

**Comment #3**

Line 342 (Power analysis): This is an "observed" power analysis. On the other hand, the specific test for which you performed this analysis is not reported. It seems that it was calculated for a repeated measures design, not for a between-within interaction. How many participants in each group would be needed to achieve a statistical power of 0.8 for detecting a medium effect size in the interaction? Provide a detailed description of this power calculation and report the software that you used.

**Response**

We have agreed and changed the description of the corresponding part. (Page 26, Lines 325-334)

“The current results deny the hypothesis set at the start of this study. However, it should be noted that the magnitude of effect size for changes in the variables having significant effect of time varied among the three training groups. It was doubtful that there is any power of detection because a total sample size of 42 participants was required according to power analysis. Due to the spread of COVID-19, however, additional subjects could not be recruited. So, the statistical power (1-β) was computed with total sample size (N = 23), effect size calculated from partial η^2^ and the significant level (p<0.05) for 1RM, muscle size, MVC, F_0_/CSA_index_ and P_max_/CSA_index_. As the results, the statistical power was more than 0.8 in all variables, indicating that the effects of the training interventions adopted in the present study had a large effect size.”
